# A Comparison of Pediatric vs. Adult Patients with the Ewing Sarcoma Family of Tumors

**DOI:** 10.3389/fonc.2017.00082

**Published:** 2017-05-08

**Authors:** Vivek Verma, Kyle A. Denniston, Christopher J. Lin, Chi Lin

**Affiliations:** ^1^Department of Radiation Oncology, University of Nebraska Medical Center, Omaha, NE, USA; ^2^Department of Radiation Oncology, St. Peter’s Health Partners, Albany, NY, USA; ^3^Creighton Preparatory School, Omaha, NE, USA

**Keywords:** Ewing sarcoma, primitive neuroectodermal tumor, pediatric oncology, survival, radiation therapy

## Abstract

**Purpose:**

This study sought to identify differences in clinical characteristics, outcomes, and treatments between adult and pediatric patients with the Ewing sarcoma family of tumors (ESFT).

**Methods:**

By using the Surveillance, Epidemiology, and End Results database from 1983 to 2013, 1,870 patients were analyzed (*n* = 976 pediatric, *n* = 894 adult). Between the two groups, demographic, tumor, and treatment characteristics were collated and compared. The chi-square test determined differences in proportions of the variables between groups. Survival analysis was performed using the Kaplan–Meier method; distributions were compared using the log-rank test. Univariate and multivariate analyses were performed to examine variables correlating with overall survival (OS), the primary endpoint.

**Results:**

Adult patients had a poorer prognosis and were more likely to present with primitive neuroectodermal tumor (PNET) histology, along with distant metastasis and soft tissue primary site. In patients undergoing surgery, radiation therapy (RT) was not associated with higher OS in either children or adults. If no surgery was performed, receipt of RT was associated with higher OS in adults but not children. Adulthood negatively correlated with OS on multivariate analysis when adjusting for potential confounding factors. Other salient factors associated with OS were male gender, metastatic disease, non-extremity bone location, treatment era, and PNET histology. However, when examining the most recent subset (patients treated from 2004 to 2013), RT was associated with improved OS in both pediatrics and adults, which was an independent predictor on multivariate analysis.

**Conclusion:**

Adult patients with ESFT have inferior survival compared to pediatric patients, likely related to earlier clinical detection in the latter.

## Introduction

The Ewing sarcoma family of tumors (ESFT) comprises a group of small, round, blue cell neoplasms that primarily affect the skeleton in adolescent children. The incidence is approximately 2.8 per million in the United States and has remained relatively stable over the past few decades (1). ESFT often amalgamates both Ewing sarcoma and primitive neuroectodermal tumors (PNETs), owing to similar histology, treatment, and outcome (2). PNETs are overall uncommon neoplasms that are thought to have a similar stem cell of origin as Ewing sarcoma, and thus, the treatment and outcomes of both are thought to be correlated (2). Both are also unified by the presence of the EWS–ETS fusion protein. However, although both Ewing sarcoma and PNET arise from neuroectoderm, PNET histopathologically display more developed cytological features of neural cells. Poor prognostic factors for ESFT include axial location, larger tumor size/volume, presence of metastatic disease, male gender, and older age (3–8).

The Ewing sarcoma family of tumors uncommonly occurs in adults, although the line that distinguishes the ages of adolescent children and adults is often blurred. Studies often define “adult” patients as 16 years or older, which may be inconsistent with other studies and/or provide a misrepresentation of the patient population (9). Currently, there are no studies using population-based databases that examine differential clinical factors between pediatric and adult cohorts. It is unlikely that prospective studies, or even comparatively large retrospective cohorts, would be able to accumulate large volumes of patients of adult ESFTs to permit robust conclusions.

Therefore, analyses of large population-based databases such as the Surveillance, Epidemiology, and End Results (SEER) database are valuable for these uncommon cases. The objective of this study was to compare clinical characteristics of pediatric (≤18 years) vs. adult (>18 years) ESFTs, impact of surgery and radiation therapy (RT) on both groups, and factors associated with overall survival (OS).

## Materials and Methods

To analyze large volumes of patients with ESFT, we utilized the SEER registry, which encompasses an estimated 28% of the US population, including minority populations (10). The patient population was assembled using the histology codes 9260, 9364, or 9365. A total of 1,870 patients from 1983 to 2013 were selected for analysis, 976 of which were pediatric cases (≤18 years) and 894 adult cases (>18 years). All the cases with missing data were included in efforts to avoid biases.

Between the two groups, demographic, tumor, and treatment characteristics were then collated and compared. Receipt of RT was coded as external-beam, radioactive implants, or radioisotopes; cancer-directed surgery referred to local tumor excision, amputation, or surgical therapy not otherwise specified. Both were similar to existing SEER publications in this tumor type (11, 12). All statistical calculations were performed using SAS version 9.4 (SAS Institute Inc., Cary, NC, USA), and *p* < 0.05 was considered statistically significant. The chi-square test was used to compare the differences in proportions for the baseline clinical characteristics between groups. Survival analysis was carried out using the Kaplan–Meier method, and distributions were compared using the log-rank test. For OS, events were defined as death from any cause. For cancer-specific survival (CSS), events were defined as death from cancer. Deaths from all other reasons and those alive at the time of analysis were censored. Univariate analysis was performed to identify factors associated with the primary endpoint, OS. Hazard ratios (HRs) and 95% confidence intervals (CIs) were calculated using Cox proportional hazards regression. To adjust for potential confounding variables, multivariate analysis was done. Only the variables positively associated with OS in the univariate analysis were elected for multivariable adjusted models (*p* ≤ 0.05 as a cutoff).

## Results

In the entire cohort (*n* = 1,870), median survival was 103 months (95% CI 78–145); 5- and 10-year OS were 55 and 49%, respectively. Median CSS was 143 months (95% CI 99–258); 5- and 10-year CSS were 57 and 51%, respectively.

Table 1 displays clinical parameters of both pediatric (*n* = 976) and adult (*n* = 894) populations. In short, adult patients were more likely to present with distant metastasis (DM), soft tissue primary site, and PNET histology.

**Table 1:** **Clinical characteristics of the entire population as well as pediatric and adult subsets**.

	Total, *N*	Pediatric, *N* (%)	Adult, *N* (%)	*p* Value
Total number	1,870	976	894	
Age				**<0.001^a^**
Median (range)	18 (0–89)	13 (0–18)	29 (19–89)	
Gender				0.936
Male	1,111	579 (59)	532 (60)	
Female	759	397 (41)	362 (40)	
Race				0.202
White	1,638	864 (89)	774 (87)	
Non-white	232	112 (11)	120 (13)	
Marital status				**<0.001**
Yes	359	3 (0)	356 (40)	
No	1,511	973 (100)	538 (60)	
SEER stage				**<0.001**
Locoregional	1,170	662 (68)	508 (57)	
Distant	545	255 (26)	290 (33)	
Missing	155	56 (6)	87 (10)	
Size				0.156
<8 cm	495	256 (52)	239 (56)	
≥8 cm	599	284 (14)	315 (16)	
Missing	776	436 (33)	340 (28)	
Primary site				**<0.001**
Extremities (bones)	556	367 (38)	189 (21)	
Axial bones	715	407 (42)	308 (35)	
Soft tissue	415	164 (17)	251 (28)	
Other	170	35 (4)	135 (15)	
Missing	14	3 (0)	11 (1)	
Lymph node				0.163
Yes	94	42 (4)	52 (6)	
No	1,052	549 (57)	503 (56)	
Missing	724	385 (39)	339 (38)	
Histology				**<0.001**
Ewing sarcoma	1,565	873 (89)	692 (77)	
PNET	305	103 (11)	202 (23)	
Grade				0.600
Low	18	8 (1)	10 (1)	
High	449	169 (17)	280 (31)	
Missing	1,403	799 (82)	604 (68)	
Year of diagnosis				**<0.001**
1983–1993	411	261 (27)	150 (17)	
1994–2003	648	309 (32)	339 (38)	
2004–2013	811	406 (41)	405 (45)	
Radiotherapy				0.054
Yes	893	490 (50)	403 (45)	
No	933	470 (48)	463 (52)	
Missing	44	16 (2)	28 (3)	
Cancer surgery				0.054
Yes	1,040	558 (57)	482 (54)	
No	675	330 (34)	345 (39)	
Missing	155	86 (9)	58 (7)	
Radiation/surgery sequence				0.894
Radiation before surgery	81	40 (4)	41 (5)	
Radiation after surgery	380	196 (20)	184 (21)	
Radiation before and after surgery	10	6 (1)	4 (0)	
No radiation or no surgery	1,396	733 (75)	663 (74)	
Missing	3	1 (0)	2 (0)	
Living status				**<0.001**
Alive	995	617 (63)	378 (42)	
Dead	875	359 (37)	516 (58)	
Cancer-specific survival				**<0.001**
Alive	995	617 (64)	378 (42)	
Cancer-specific death	784	324 (33)	460 (52)	
Other death	53	19 (2)	34 (4)	
Unknown death	38	9 (1)	17 (2)	

*^a^Wilcoxon rank-sum test utilization instead of chi-square test. Missing data were not included for p value calculations*.

*SEER, surveillance, epidemiology, and end results; PNET, primitive neuroectodermal tumor*.

*Bold means statistically significant (p < 0.05)*.

**Figure 1:**
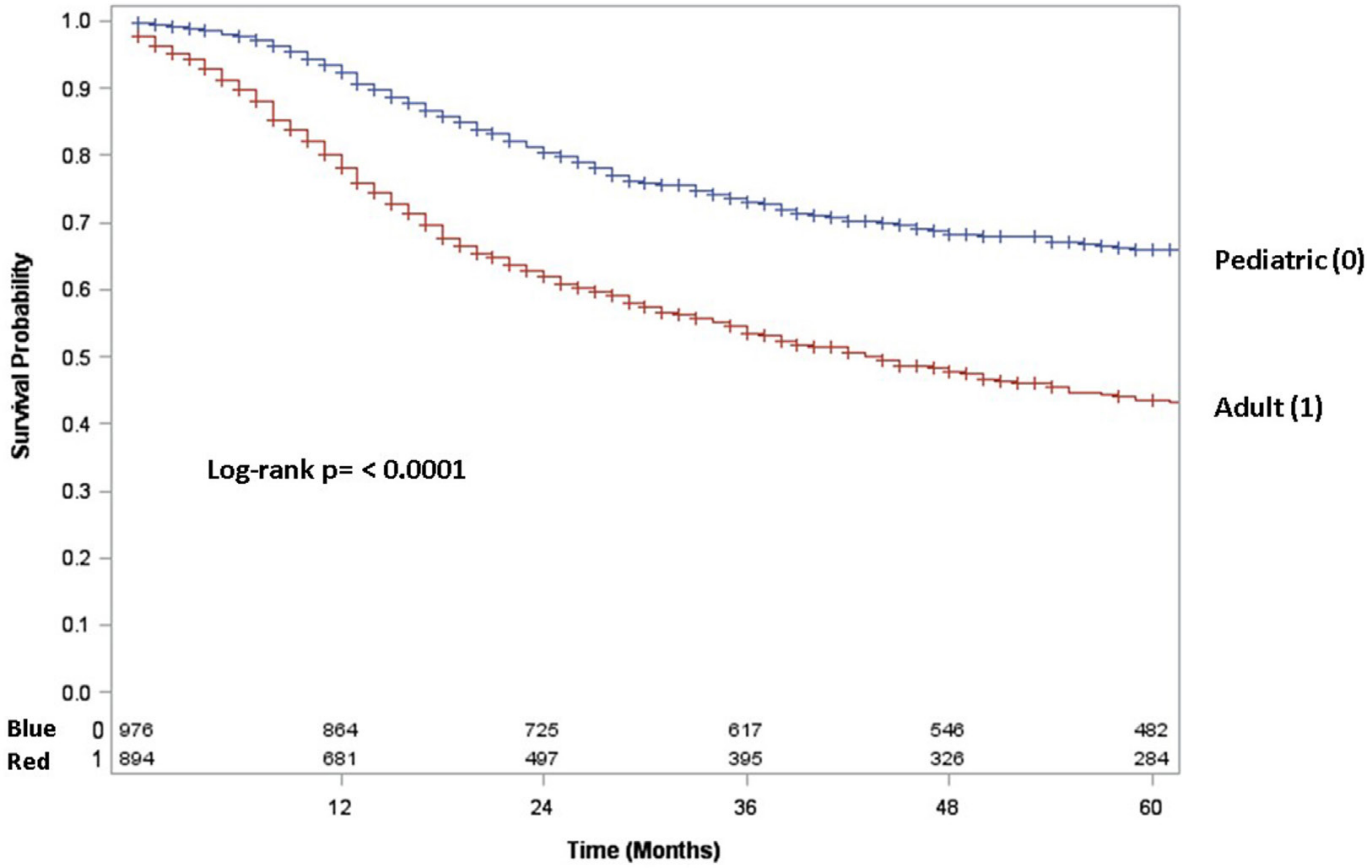
**Overall survival in pediatric (blue line) vs. adult (red line) patients**.

**Figure 2:**
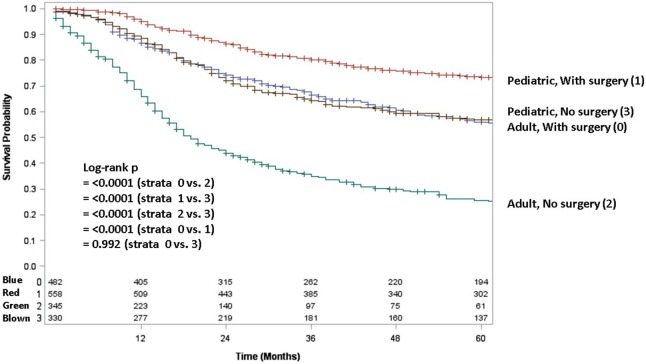
**Overall survival in pediatric and adult patients as stratified by receipt of surgery**. Red line denotes pediatric patients undergoing surgery; brown line pediatric patients without surgery; blue line adult patients undergoing surgery; green line adult patients without surgery.

**Figure 3:**
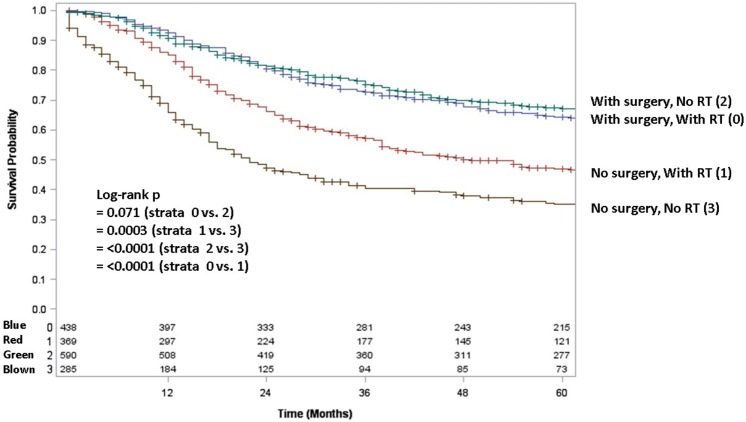
**Overall survival in pediatric and adult patients with and without surgery as stratified by receipt of radiotherapy**. Green line denotes surgery alone; blue line surgery and radiotherapy; red line radiotherapy alone; brown line neither surgery nor radiotherapy.

Adult patients with ESFT had a worse prognosis (5-year OS of 43% for adult vs. 66% for pediatrics, log-rank *p* < 0.001) (Figure 1). In both patient cohorts, surgery was associated with a large magnitude of OS improvement (Figure 2). When stratified by receipt of surgery, adults had worse 5-year OS, both with (56 vs. 73%, log-rank *p* < 0.001) and without surgery (25 vs. 57%, log-rank *p* < 0.001) (Figure 2). In patients without surgery, RT was associated with improved OS (Figure 3). However, in patients who had surgery, RT failed to improve OS (Figure 3).

Table 2 displays univariate and multivariate analyses of factors associated with OS in the whole cohort. Adulthood conferred an independent association with worse OS on multivariate analysis. Other salient factors associated with OS were male gender, metastatic disease, non-extremity bone location, and PNET histology. Of note, receipt of RT was not correlated in itself or with respect to surgery. In addition, OS was increased with diagnosis/treatment in recent years compared to the past, likely owing to better techniques and therapies. When examining pediatric (Table 3) and adult (Table 4) patients separately with multivariate analysis of factors associated with OS, similar parameters were identified in both groups. Notably, gender was a factor in adult but not pediatric patients.

**Table 2:** **Univariate and multivariate analyses of factors associated with overall survival**.

Parameter	*N*	Univariate		Multivariate	
		HR (95% CI)	*p* Value	HR (95% CI)	*p* Value
Age >18 vs. ≤18	894/976	2.062 (1.800–2.363)	**<0.001**	1.869 (1.622–2.154)	**<0.001**
Gender: male vs. female	1,111/759	1.207 (1.051–1.385)	**0.008**	1.211 (1.054–1.392)	**0.007**
Race: non-white vs. white	232/1,638	1.179 (0.966–1.439)	0.105		
Stage: metastatic vs. non-metastatic	545/1,170	3.181 (2.763–3.663)	**<0.001**	2.671 (2.301–3.100)	**<0.001**
Primary tumor site: axial bones vs. extremity bones	715/556	1.515 (1.279–1.795)	**<0.001**	1.227 (1.032–1.460)	**0.021**
Primary tumor site: soft tissue vs. extremity bones	415/556	1.460 (1.200–1.778)	**<0.001**	1.301 (1.041–1.625)	**0.021**
Primary tumor site: other vs. extremity bones	170/556	2.202 (1.730–2.802)	**<0.001**	1.500 (1.134–1.985)	**0.005**
Histology: PNET vs. Ewing	305/1,565	1.521 (1.287–1.796)	**<0.001**	1.302 (1.064–1.593)	**0.011**
Year of diagnosis: 1993–2002 vs. 1983–1992	648/411	0.986 (0.836–1.163)	0.867	0.805 (0.673–0.964)	**0.019**
Year of diagnosis: 2003–2013 vs. 1983–1992	811/411	0.799 (0.670–0.954)	**0.013**	0.703 (0.582–0.849)	**<0.001**
Radiotherapy: yes vs. no	893/933	1.058 (0.925–1.212)	0.411	0.922 (0.801–1.060)	0.252
Cancer surgery: yes vs. no	1,040/675	0.477 (0.414–0.549)	**<0.001**	0.591 (0.509–0.687)	**<0.001**
Radiotherapy after surgery vs. prior to surgery	380/81	1.026 (0.712–1.477)	0.892		

*HR, hazard ratio; CI, confidence interval; PNET, primitive neuroectodermal tumor*.

*Bold means statistically significant (p < 0.05)*.

**Table 3:** **Univariate and multivariate analyses of factors associated with overall survival in pediatric patients**.

Parameter	*N*	Univariate		Multivariate	
		HR (95% CI)	*p* Value	HR (95% CI)	*p* Value
Gender: male vs. female	579/397	1.159 (0.935–1.437)	0.178		
Race: non-white vs. white	112/864	0.972 (0.634–1.490)	0.896		
Stage: metastatic vs. non-metastatic	255/662	3.668 (2.864–4.697)	**<0.001**	2.763 (2.384–3.201)	**<0.001**
Primary tumor site: axial bones vs. extremity bones	407/367	1.696 (1.303–2.208)	**<0.001**	1.281 (1.081–1.518)	**0.004**
Primary tumor site: soft tissue vs. extremity bones	164/367	1.221 (0.817–1.826)	0.330	1.593 (1.303–1.947)	**<0.001**
Primary tumor site: other vs. extremity bones	35/367	1.820 (0.742–4.467)	0.191	2.107 (1.649–2.692)	**<0.001**
Histology: PNET vs. Ewing	103/873	1.235 (0.708–2.152)	0.457		
Year of diagnosis: 1994–2003 vs. 1983–1993	309/261	0.645 (0.475–0.877)	**0.005**	0.913 (0.771–1.081)	0.292
Year of diagnosis: 2004–2013 vs. 1983–1993	405/261	0.439 (0.294–0.656)	**<0.001**	0.768 (0.641–0.920)	**0.004**
Radiotherapy: yes vs. no	490/470	1.460 (1.174–1.816)	**0.001**	0.882 (0.769–1.012)	0.073
Cancer surgery: yes vs. no	558/330	0.533 (0.423–0.671)	**<0.001**	0.584 (0.507–0.672)	**<0.001**
Radiotherapy after surgery vs. prior to surgery	196/40	0.710 (0.426–1.186)	0.191		

*HR, hazard ratio; CI, confidence interval; PNET, primitive neuroectodermal tumor*.

*Bold means statistically significant (p < 0.05)*.

**Table 4:** **Univariate and multivariate analyses of factors associated with overall survival in adult patients**.

Parameter	*N*	Univariate		Multivariate	
		HR (95% CI)	*p* Value	HR (95% CI)	*p* Value
Gender: male vs. female	532/362	1.259 (1.052–1.507)	**0.012**	1.213 (1.056–1.393)	**0.006**
Race: non-white vs. white	120/774	1.189 (0.923–1.531)	0.181		
Marital status: yes vs. no	356/538	1.160 (0.973–1.382)	0.098		
Stage: metastatic vs. non-metastatic	290/508	2.960 (2.454–3.570)	**<0.001**	2.749 (2.371–3.187)	**<0.001**
Primary tumor site: axial bones vs. extremity bones	308/189	1.334 (1.041–1.710)	**0.023**	1.253 (1.058–1.484)	**0.009**
Primary tumor site: soft tissue vs. extremity bones	251/189	1.325 (1.021–1.719)	**0.034**	1.379 (1.111–1.711)	**0.004**
Primary tumor site: other vs. extremity bones	135/189	1.773 (1.324–2.374)	**0.001**	1.811 (1.386–2.366)	**<0.001**
Histology: PNET vs. Ewing	202/692	1.618 (1.332–1.964)	**<0.001**	1.315 (1.079–1.603)	**0.007**
Year of diagnosis: 1994–2003 vs. 1983–1993	339/150	1.207 (0.913–1.596)	0.187		
Year of diagnosis: 2004–2013 vs. 1983–1993	405/150	1.098 (0.788–1.531)	0.581		
Radiotherapy: yes vs. no	403/463	0.828 (0.694–0.989)	**0.037**	0.882 (0.769–1.012)	0.073
Cancer surgery: yes vs. no	482/345	0.427 (0.356–0.513)	**<0.001**	0.591 (0.510–0.686)	**<0.001**
Radiotherapy after surgery vs. prior to surgery	184/41	1.446 (0.858–2.437)	0.166		

*HR, hazard ratio; CI, confidence interval; PNET, primitive neuroectodermal tumor*.

*Bold means statistically significant (p < 0.05)*.

**Figure 4:**
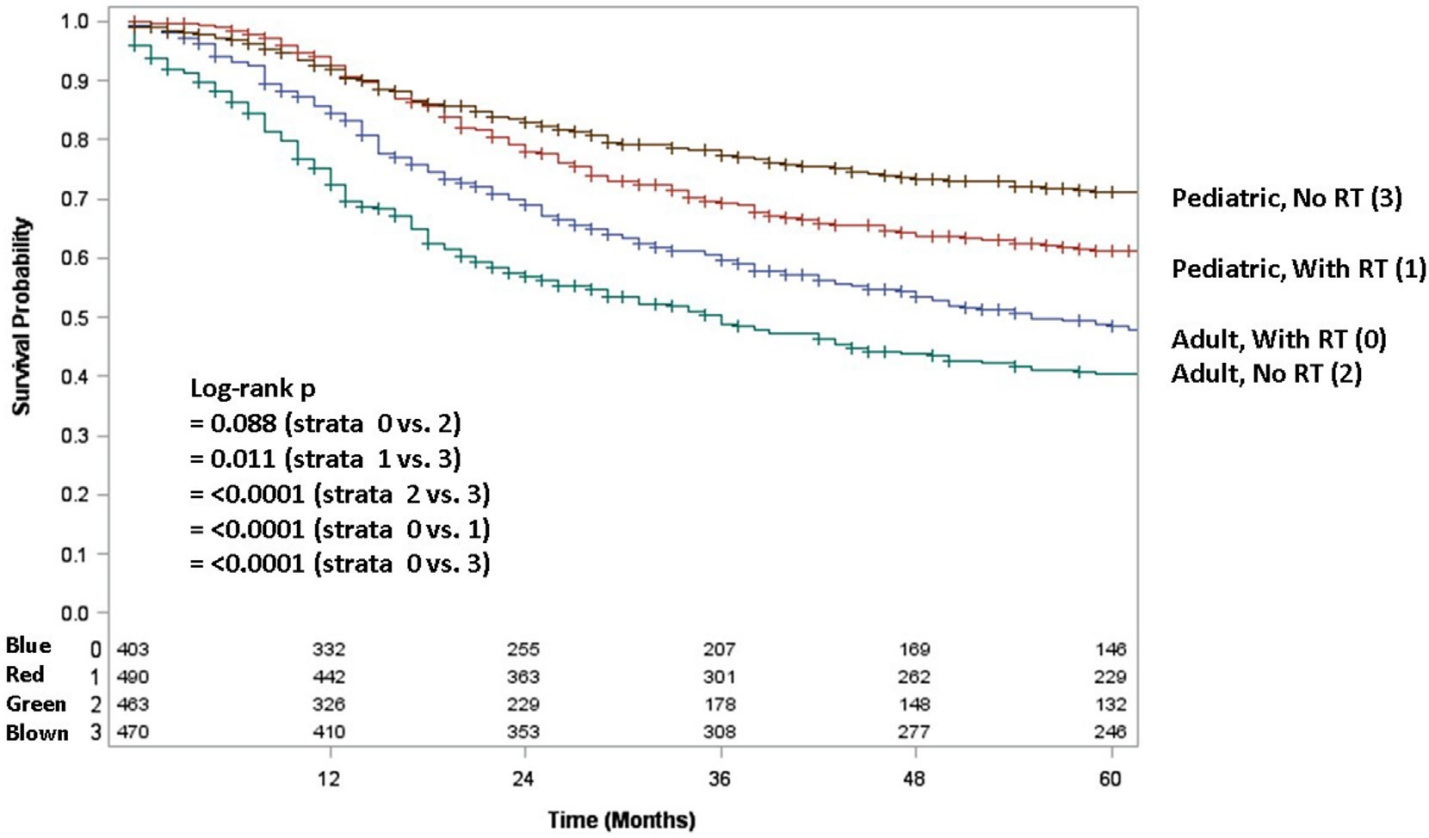
**Overall survival in pediatric and adult patients without surgery as stratified by receipt of radiotherapy**. Brown line denotes pediatric patients without radiotherapy; red line pediatric patients undergoing radiotherapy; blue line adult patients undergoing radiotherapy; green line adult patients without radiotherapy.

**Figure 5:**
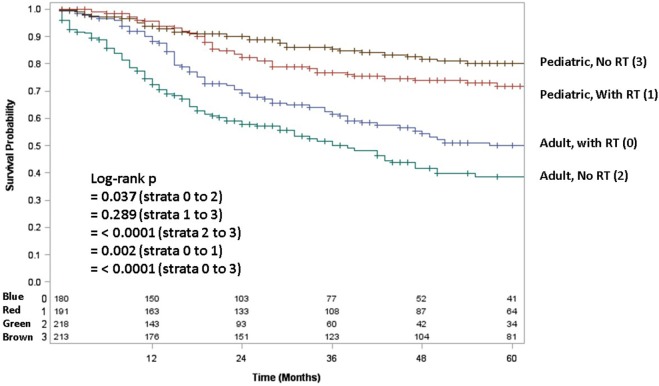
**In the 2004–2013 cohort, overall survival in pediatric and adult patients as stratified by receipt of radiotherapy**. Brown line denotes pediatric patients without radiotherapy; red line pediatric patients undergoing radiotherapy; blue line adult patients undergoing radiotherapy; green line adult patients without radiotherapy.

Because testing for the EWS/FLI translocation became available in the mid to late 1990s, we sought to further investigate the subset treated in the most recent decade (2004–2013), during which the most modern paradigms of diagnosis and treatment were most likely to be utilized. Table 5 (analogous to Table 1) displays similar comparisons between pediatric and adult groups, although notably, receipt of RT was no longer statistically significant. When comparing survival in this cohort, there were similar conclusions (e.g., adults had poorer prognosis and surgery improved survival), with one exception. In the overall cohort of patients (Figure 4), RT did not improve OS in adults and was associated with worse OS in pediatrics; the same was not true in the most modern subset (Figure 5). There was no statistical difference in OS with or without RT in pediatric patients; moreover, adults receiving RT had such with a significant improvement in OS. Tables 6–8 (analogous to Tables 2–4) illustrate that RT was independently associated with OS in all patients as well as adult and pediatric subsets separately. Of note, this difference was observed only on multivariate analysis (non-significant on univariate analysis), potentially relating to interaction with factors that were significant on univariate but non-significant on multivariate analysis (e.g., some comparisons of primary site).

**Table 5:** **Clinical characteristics of the entire population from 2004 to 2013 as well as pediatric and adult subsets**.

	Total, *N*	Pediatric, *N* (%)	Adult, *N* (%)	*p* Value
Total number	811	406	405	
Age				**<0.001^a^**
Median (range)	18 (0–89)	13 (0–18)	29 (19–89)	
Gender				0.700
Male	474	240 (59)	234 (58)	
Female	337	166 (41)	171 (42)	
Race				0.684
White	689	347 (85)	342 (84)	
Non-white	122	59 (15)	63 (16)	
Marital status				**<0.001**
Yes	146	2 (0.5)	144 (36)	
No	665	404 (99.5)	261 (64)	
SEER stage				**0.007**
Locoregional	511	279 (69)	232 (57)	
Distant	241	106 (26)	135 (33)	
Missing	59	21 (5)	38 (10)	
Size				**0.045**
<8 cm	250	134 (33)	116 (29)	
≥8 cm	318	144 (35)	174 (43)	
Missing	243	128 (32)	215 (28)	
Primary site				**<0.001**
Extremities (bones)	212	132 (33)	80 (20)	
Axial bones	293	162 (40)	131 (32)	
Soft tissue	213	91 (22)	122 (30)	
Other	87	20 (5)	67 (17)	
Missing	6	1 (0.3)	5 (1)	
Lymph node				**0.637**
Yes	61	30 (7)	31 (8)	
No	642	336 (83)	306 (75)	
Missing	108	40 (10)	68 (17)	
Histology				**<0.001**
Ewing sarcoma	676	361 (89)	315 (78)	
PNET	135	45 (11)	90 (22)	
Grade				**0.622**
Low	9	4 (1)	5 (1)	
High	168	61 (15)	107 (27)	
Missing	634	341 (84)	293 (72)	
Year of diagnosis				0.113
2004–2008	387	205 (50)	182 (45)	
2009–2013	424	201 (50)	223 (55)	
Radiotherapy				0.560
Yes	371	191 (47)	180 (44)	
No	431	213 (52.5)	218 (54)	
Missing	9	2 (0.5)	7 (2)	
Cancer surgery				**0.014**
Yes	489	265 (65)	224 (55)	
No	301	136 (34)	165 (41)	
Missing	21	5 (1)	16 (4)	
Radiation/surgery sequence				0.169
Radiation before surgery	28	9 (2)	19 (4.7)	
Radiation after surgery	179	94 (23)	85 (21)	
Radiation before and after surgery	1	0 (0)	1 (0.25)	
No radiation or no surgery	602	303 (75)	299 (73.8)	
Missing	1	0 (0)	1 (0.25)	
Living status				**<0.001**
Alive	536	318 (78)	218 (54)	
Dead	275	88 (22)	187 (46)	
Cancer-specific survival				**<0.001**
Alive	536	318 (78.3)	218 (54)	
Cancer-specific death	255	83 (20.4)	172 (42)	
Other death	11	4 (1)	7 (2)	
Unknown death	9	1 (0.3)	8 (2)	

*^a^Wilcoxon rank-sum test utilization instead of chi-square test. Missing data were not included in p value calculations*.

*SEER, surveillance, epidemiology, and end results; PNET, primitive neuroectodermal tumor*.

*Bold means statistically significant (p < 0.05)*.

**Table 6:** **Of the 2004–2013 cohort, univariate and multivariate analyses of factors associated with overall survival**.

Parameter	*N*	Univariate		Multivariate	
		HR (95% CI)	*p* Value	HR (95% CI)	*p* Value
Age >18 vs. ≤18	405/406	3.835 (2.565–5.732)	**<0.001**	2.367 (1.812–3.093)	**<0.001**
Gender: male vs. female	474/337	1.109 (0.869–1.415)	0.405		
Race: non-white vs. white	122/689	1.423 (1.043–1.940)	**0.026**		
Stage: metastatic vs. non-metastatic	241/511	3.328 (2.185–5.067)	**<0.001**	4.056 (3.071–5.358)	**<0.001**
Primary tumor site: axial bones vs. extremity bones	293/212	1.655 (1.176–2.327)	**0.004**	1.258 (0.884–1.788)	0.202
Primary tumor site: soft tissue vs. extremity bones	213/212	1.729 (1.203–2.485)	**0.003**	1.417 (0.958–2.098)	0.081
Primary tumor site: other vs. extremity bones	87/212	2.566 (1.684–3.908)	**<0.001**	1.422 (0.906–2.231)	0.126
Histology: PNET vs. Ewing	135/676	1.786 (1.352–2.358)	**<0.001**	1.443 (1.058–1.968)	**0.021**
Radiotherapy: yes vs. no	371/431	0.960 (0.756–1.219)	0.739	0.776 (0.604–0.998)	**0.048**
Cancer surgery: yes vs. no	489/301	0.351 (0.275–0.448)	**<0.001**	0.519 (0.399–0.676)	**<0.001**
Radiotherapy after surgery vs. prior to surgery	179/28	1.150 (0.554–2.384)	0.708		

*HR, hazard ratio; CI, confidence interval; PNET, primitive neuroectodermal tumor*.

*Bold means statistically significant (p < 0.05)*.

**Table 7:** **Of the 2004–2013 cohort, univariate and multivariate analyses of factors associated with overall survival in pediatric patients**.

Parameter	*N*	Univariate		Multivariate	
		HR (95% CI)	*p* Value	HR (95% CI)	*p* Value
Gender: male vs. female	240/166	0.990 (0.495–1.981)	0.977		
Race: non-white vs. white	59/347	2.061 (0.897–4.733)	0.088		
Stage: metastatic vs. non-metastatic	106/279	7.932 (4.416–14.25)	**<0.001**	4.066 (3.094–5.343)	**<0.001**
Primary tumor site: axial bones vs. extremity bones	162/132	2.172 (1.194–3.950)	**0.011**	1.234 (0.878–1.733)	0.226
Primary tumor site: soft tissue vs. extremity bones	91/132	2.137 (0.952–4.798)	0.066	1.767 (1.236–2.525)	**0.002**
Primary tumor site: other vs. extremity bones	20/132	5.715 (1.161–28.14)	**0.032**	2.200 (1.456–3.326)	**<0.001**
Histology: PNET vs. Ewing	45/361	1.416 (0.553–3.629)	0.469		
Radiotherapy: yes vs. no	191/213	1.181 (0.589–2.368)	0.639	0.771 (0.601–0.989)	**0.040**
Cancer surgery: yes vs. no	265/136	0.409 (0.216–0.773)	**0.006**	0.474 (0.366–0.615)	**<0.001**
Radiotherapy after surgery vs. prior to surgery	94/9	1.298 (0.300–5.624)	0.727		

*HR, hazard ratio; CI, confidence interval; PNET, primitive neuroectodermal tumor*.

*Bold means statistically significant (p < 0.05)*.

**Table 8:** **Of the 2004–2013 cohort, univariate and multivariate analyses of factors associated with overall survival in adult patients**.

Parameter	*N*	Univariate		Multivariate	
		HR (95% CI)	*p* Value	HR (95% CI)	*p* Value
Gender: male vs. female	234/171	1.149 (0.855–1.544)	0.358		
Race: non-white vs. white	63/342	1.288 (0.875–1.897)	0.200		
Stage: metastatic vs. non-metastatic	135/232	3.915 (2.848–5.382)	**<0.001**	4.108 (3.126–5.398)	**<0.001**
Primary tumor site: axial bones vs. extremity bones	131/80	1.422 (0.910–2.222)	0.123	1.214 (0.863–1.706)	0.265
Primary tumor site: soft tissue vs. extremity bones	122/80	1.376 (0.874–2.167)	0.168	1.463 (0.996–2.151)	0.053
Primary tumor site: other vs. extremity bones	67/80	1.686 (1.025–2.775)	**0.040**	1.874 (1.216–2.887)	**0.004**
Histology: PNET vs. Ewing	90/315	1.577 (1.145–2.171)	**0.005**	1.573 (1.151–2.151)	**0.005**
Radiotherapy: yes vs. no	180/218	0.709 (0.528–0.951)	**0.022**	0.763 (0.594–0.979)	**0.034**
Cancer surgery: yes vs. no	224/165	0.330 (0.243–0.446)	**<0.001**	0.489 (0.377–0.634)	**<0.001**
Radiotherapy after surgery vs. prior to surgery	85/19	1.291 (0.544–3.062)	0.562		

*HR, hazard ratio; CI, confidence interval; PNET, primitive neuroectodermal tumor*.

*Bold means statistically significant (p < 0.05)*.

## Discussion

Despite some small studies that have claimed no differences between the behavior of ESFTs in children and adults (2), the use of a national database with large volumes determines that there are important differences. We determine clinical characteristics that are differentially associated with adult ESFT, describe which populations radiotherapy may benefit, and describe parameters associated with OS in ESFTs regardless of age.

Our results are similar to other data in that metastatic disease, treatment era, location, gender, and age are linked with OS (3–8). Although our study details characteristics of adult and pediatric subpopulations in a high-volume manner, work with smaller sample sizes has confirmed that prognosis in adults is poorer (13). We demonstrate that receipt of surgery is of utmost importance, whereas RT was not associated with improved outcomes in the pediatric population. It is, however, likely that there are several other pieces of information that may explain these findings. First, the lack of chemotherapy information and time to treatment are major limitations of the SEER database, and some have posited that the higher doses of chemotherapy given to children as well as earlier treatment initiation may substantially impact outcomes (13). Second, it is also likely that those patients receiving RT in any capacity may be a preselected population more likely to have poorer disease characteristics such as larger tumor size, unresectable location, and/or incomplete resection. As such, a proper comparison of both modalities remains undefined. Third, because adults were more likely to have metastatic disease and larger tumors, it is also likely that these tumors are clinically detected earlier in children than adults.

Additionally, it is compelling that the most recent subset (2004–2013) disputes many of the conclusions made in the general population of patients, indicating that perhaps modern treatment paradigms may select for RT patients in a better manner, together with improved surgical techniques and potentially even systemic therapy.

Supporting these data is institutional work from the Univer-sity of Toronto (13) analyzing 53 patients receiving VAC-IE che-motherapy. The goal of the study is to compare outcomes between pediatric (*n* = 29, defined as <18 years) and adult (*n* = 24, ≥18 years) cohorts. Adult patients, who experienced worse OS, tended to receive lower doses of IE chemotherapy and received local therapy at a later time point than pediatric counterparts; the latter independently predicted for OS on multivariate analysis. Hence, although the authors concluded that adults have poorer OS than children, the report served to show that other factors not assessed by the majority of this and similar studies (e.g., time to local therapy) could be novel prognostic factors for survival.

Overall, prognostic factors in adults and children seem to be a difficult issue to address (3, 6). In adults, these included metastasis at diagnosis and pelvic primary tumor. In children, stage independently predicted survival; larger tumors and disease at axial locations were more likely to present with metastatic disease. Thus, although mirrored well by this study in context of other data, in none of these studies has causation been implied; likely, there are an interconnected set of factors that collectively lead to poor prognosis.

There are several limitations of our analysis. In addition to the inherently retrospective nature of SEER studies as well as individualized follow-up, it must be once again prominently mentioned that causation can neither be stated nor implied with these data, especially regarding treatment interventions and survival. The SEER database also does not allow for information regarding chemotherapy, surgical margins, pathological confirmation (e.g., identifying whether a few tumors were indeed low-grade Ewing sarcomas, which would be exceedingly rare), and radiation doses. In addition, the missing values for several parameters in Table 1 prevent robust conclusions even though the groups may differ based on statistical tests (likely owing to the number of unknown values). In addition, selection bias for any patient receiving surgery (or extent of surgery) can never be ruled out, as mentioned above. Moreover, confounding items such as era of treatment (especially since newer paradigms independently correlate with increased OS) are a necessary limitation that must be accepted to accumulate sufficient sample sizes. Even the inclusion of patients treated a decade ago likely encompasses a cohort with worse OS than those treated in the present decade. The diagnosis of Ewing sarcoma based on EWS/FLI translocation becoming available in the mid-1990s may also be a confounder, but to assure high volumes of patients concordant with prior studies, a facet unique to the SEER database, we opted to include all treatment eras (7, 11, 12).

Going forward, it must be recognized that there are age-based subgroups of adult and pediatric cohorts that may offer further elucidation. For instance, the majority of the adult cohort in this study was predictably skewed toward younger patients, with few who were of middle and advanced age. The role of various treatment paradigms in these subgroups is uncertain. Similarly, the infant (<12 months) subgroup has been studied as another example, determining a potential increase in early death but similar OS (14). In addition, studying a large-volume cohort of ESFTs treated in the present decade using the most modern surgical techniques, chemotherapy regimens, and radiotherapy technologies [including proton beam therapy (15, 16)] is of great necessity to determine that the results presented herein are accurate and representative of modern treatment paradigms.

## Conclusion

In our high-volume comparison of pediatric vs. adult ESFT patients, adult patients had a poorer prognosis and were more likely to present with PNET histology, along with DM and soft tissue primary site. When adjusting for potential confounders on multivariate analysis, adult patients were independently associated with worse OS, along with male gender, metastatic disease, non-extremity bone location, treatment era, and PNET histology.

## Ethics Statement

This study was deemed exempt from the Institutional Review Board of the University of Nebraska Medical Center and Ethics Committee on account of no patient identifiers in a national, public database; there were no patients participating in the study.

## Author Contributions

KD obtained data, CJL performed data analysis, VV wrote the manuscript, and CL conceived of the study and performed supervisory roles. All the authors read, reviewed, and approved the manuscript.

## Conflict of Interest Statement

The authors declare that the research was conducted in the absence of any commercial or financial relationships that could be construed as a potential conflict of interest.
